# Assessing Frontline Personnel’s Recognition of and Response to Cases of Abuse in Later Life

**DOI:** 10.1093/geroni/igab046.2293

**Published:** 2021-12-17

**Authors:** Sarah Marrs, Courtney O'Hara, Ruth Anne Young, Miranda Yelvington, Deijah Patterson, Annie Rhodes, Edward Ansello

**Affiliations:** Virginia Commonwealth University, Richmond, Virginia, United States

## Abstract

Abuse in later life is experienced by 10% of adults over age 60 (Acierno et al., 2010). Unfortunately, it is estimated that for every one reported case, around 20 remain unreported (APA, 2012). A primary reason for this disparity is the absence of training provided to professionals working at the frontline of elder abuse, such as law enforcement professionals, health care professionals, and aging and victim service providers (e.g., Rose et al., 2016). This leaves the workforce best positioned to intervene in cases of abuse in later life lacking knowledge around what constitutes the different types of abuse and what they should do if they suspect abuse (Rosen et al., 2018). A critical first step to developing the evidence-based training needed to reconcile this gap is to gain a better understanding of the current landscape within this workforce. This qualitative study explored the knowledge and attitudes towards abuse in later life as well as current practices and policies for reporting abuse among law enforcement professionals (n = 1), health care professionals (n = 2), and aging (n = 5) and victim (n = 4) service providers. Participants (N = 12) represented urban (n = 4), suburban (n = 5), and rural settings (n = 2). Themes emerging from the focus groups highlight a number of barriers to identifying and reporting abuse for professionals in each discipline. Our findings also provide strong evidence of the need to intervene and diminish the impact ageist attitudes and behaviors can have on older adults.

